# Gold Nanoparticle-Mediated Lateral Flow Assays for Detection of Host Antibodies and COVID-19 Proteins

**DOI:** 10.3390/nano12091456

**Published:** 2022-04-25

**Authors:** Leila Safaee Ardekani, Peter Waaben Thulstrup

**Affiliations:** 1 Department of Nanobiotechnology, Faculty of Biological Sciences, Tarbiat Modares University, Tehran P.O. Box 14115-111, Iran; 2 Department of Chemistry, University of Copenhagen, Universitetsparken 5, DK-2100 Copenhagen, Denmark

**Keywords:** SARS-CoV-2, lateral flow assay, antigen test, antibody test, gold nanoparticle, immunochromatography

## Abstract

Coronaviruses, that are now well-known to the public, include a family of viruses that can cause severe acute respiratory syndrome (SARS) and other respiratory diseases, such as Middle East respiratory syndrome (MERS). Severe acute respiratory syndrome coronavirus 2 (SARS-CoV-2), the seventh member of this coronavirus family, was detected in 2019 and can cause a number of respiratory symptoms, from dry cough and fever to fatal viral pneumonia. Various diagnostic assays ranging from real-time polymerase chain reaction (RT-PCR) to point-of-care medical diagnostic systems have been developed for detection of viral components or antibodies targeting the virus. Point-of-care assays allow rapid diagnostic assessment of infectious patients. Such assays are ideally simple, low-cost, portable tests with the possibility for on-site field detection that do not require skilled staff, sophisticated equipment, or sample pretreatment, as compared to RT-PCR. Since early 2021 when new SARS-CoV-2 variants of concern increased, rapid tests became more crucial in the disease management cycle. Among rapid tests, gold nanoparticle (GNP)-based lateral flow assays (LFAs) have high capacity for performing at the bedside, paving the way to easy access to diagnosis results. In this review, GNP-based LFAs used for either COVID-19 proteins or human response antibodies are summarized and recommendations for their improvement have been suggested.

## 1. Introduction

The COVID-19 pandemic has affected people’s lives around the world. The SARS-CoV-2 pathogen, which belongs to the ß-coronavirus family, has four structural proteins, namely the envelope (E), membrane (M), spike (S), and nucleocapsid (N) proteins. The S protein contains three parts: a large ectodomain, a single-pass transmembrane anchor, and a short intracellular tail. The ectodomain involved in viral entry into host cells via binding to the angiotensin-converting enzyme 2 (ACE-2) receptor contains a receptor-binding domain (RBD) subunit S1 and a membrane-fusion subunit S2 [1].

The symptoms of infection vary widely, from mild cough to severe lung injuries. The diagnosis of COVID-19 infection is based on either the detection of the virus genes and proteins, or parts of the host-infected cells and immunological reactions [2].

So far, a variety of biosensors ranging from colorimetric-based assays to electrochemical sensors [3,4,5,6,7,8,9,10,11,12,13,14,15,16] have been used for the detection of COVID-19 infection. Table 1 shows the different targets and methods which have been used for COVID-19 diagnosis.

## 2. Lateral Flow Assays

**Figure 1:**
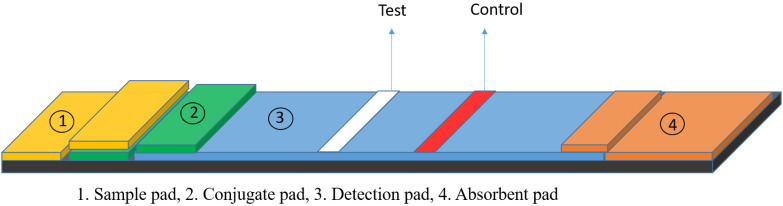
Schematic overview of a lateral flow assay (LFA) device showing the four components and the test and control lines for readout of the result.

An immunochromatographic strip or a lateral flow assay is one type of microfluidic assay in which the sample is transported along the test strip by capillary force. It is typically paper-based and consists of a sample pad, a conjugate pad, a detection pad, and an absorbent or wicking pad (Figure 1). The components of an LFA have been explained in more detail in some reviews [17,18]. Generally, there are two formats of lateral flow tests, namely the competitive and the sandwich assays [19]. In the sandwich format, the color intensity at the test line has a linear correlation to the concentration of the target analyte, while in the competitive format, the appearance of color at the test line indicates a negative result [20].

Lateral flow assays (LFAs) have also been employed for detection of SARS-CoV-2, and these can be classified into three groups: RNA tests, host immune response detection, and viral surface detection.

## 3. GNP-Based LFAs

A variety of signal reporters have been applied based on different technologies, e.g., europium-chelate-based fluorescent nanoparticles [21], Cy5 [22], FAM [23], lanthanide [24], selenium [25], and cellulose nanobeads [26,27] have, for example, been used in the LFA format for COVID-19 diagnosis. Among colored reporters, gold nanoparticles (Au-NPs) with high color intensity can lead to enhanced sensitivity in an LFA [17]. Moreover, other features of gold nanoparticles such as nontoxicity, cost effectiveness, ease of production, stability in dried form, and facile conjugation with biomolecules make them the ideal candidates to use as signal reporter and suitable for industrial application [28]. Au-NPs exhibit strong color in the visible region due to plasmon surface effects, and they can readily be conjugated with biomolecules such as antibodies to achieve a desired biosensor design. Au-NP-based lateral flow assays have been employed for detection of a significant number of pathogens from influenza viruses [29] to more potent bacteria such as *E.coli* and other pathogens [30,31,32,33].

GNP-based LFAs detection of COVID-19 are designed to detect either the viral RNA or proteins or antibodies to SARS-CoV-2. CRISPR-based assays such as DETECTR, FELUDA, SHERLOCK, AIOD-CRISPR, ENHANCE, and CASdetect, which are more sensitive than the detection of antibodies and COVID-19 antigens, have also been reported for detection of RNA [34].

CRISPR-Cas 9-mediated LFA [24] is an example of a developed gold-based LFA for detection of COVID-19 RNA. In this process, the gene-encoding envelope (E) and open-reading frame 1ab (ORF1ab) are amplified by tagged primers with reverse transcription-recombinase polymerase amplification (RT-RPA). Cas9/sgRNA complexes specifically recognize the tagged amplicons. Then, attachment of GNP-DNA probes on sgRNA and their accumulation on the test lines lead to visual signals and show the presence of the target analyte in the sample [21].

The integration of the DETECTR platform and the advantages of the LFA format has been introduced by Mammoth Biosciences Inc. as an alternative for the early and visual detection of genetic material of viruses with high sensitivity and specificity [34]. Detection of conserved area of the SARS-CoV-2 genome using a combination of the CRISPR-ENHANCE technique and LFA was performed by Jain et al. [35]. Detection of SARS-CoV-2 and its circulating RNA variants can be performed by a CRISPR-based LFA [36].

Although these kinds of assays have shown great specificity and sensitivity, the need for purification steps and instrument hardware make them unsuitable for point-of-care detection [34,35].

Diagnostic methods for detection of COVID-19 have been discussed in several reviews. For example, Singhal et al. have summarized nanotechnology-based biosensors and categorized them based on different bio-recognition elements [37]. Laboratory-based detection methods ranging from cell culture-based detection to immunological detection have been discussed by Febrraio et al. [2]. In other studies, methods available for COVID-19 detection ranging from chest computerized tomography (CT) to biosensors have been reported [38,39]. Hong et al. have discussed some considerations for developing GNP-based biosensors for SARS-CoV-2 diagnosis [40]. While these papers briefly talk about different methods for detection of COVID-19, there are studies that specifically focus on LFAs that have more potential for commercialization.

For example, some reviews have summarized LFAs for COVID-19 diagnostics [41], and others have introduced some solutions for improving the performance of LFAs for COVID-19 detection [42,43]. In the present review, we discuss the recent advances in GNP-based LFAs for COVID-19 that have higher potential of gaining worldwide use even outside of urbanized areas in lesser developed regions. The development of each assay along with its strengths and weakness are discussed. We also review several strategies that have been used for enhancing the sensitivity and specificity of GNP-based LFAs with application to COVID-19 and discuss other methods that can be applied for further improving COVID-19 diagnosis via GNP-based LFAs, as well as some of the potential challenges to be addressed.

We applied the following search terms: (lateral flow assay or immunochromatographic strip or test strip) and (gold nanoparticle) and (antigen test or antibody test) and (coronavirus or COVID-19 or SARS-CoV-2) across several databases, such as PubMed(Rockville Pike, Bethesda, MD, USA), Elsevier(Amsterdam, The Netherlands), Science Direct (Amsterdam, The Netherlands), and Google Scholar (Mountain View, CA, USA), for relevant published studies on 1 March 2022. Among 3626 items, we selected 112 publications in which gold-based LFA technology has been used for the detection of viral antigens or antibodies to SARS-CoV-2.

## 4. Gold-Based LFA for COVID-19 Diagnosis

### 4.1. Detection of Host Antibody

Serological tests that measure immune responses are suitable for broad seroprevalence surveys in an affected area, to identify potential plasma donors, screen asymptomatic persons, for vaccine evaluation, and to find sociological and geographic relation to mitigate the epidemic [44]. In contrast to a molecular assay, antibody-based methods have higher accuracy because of a more uniform antibody distribution in blood compared to viral loading in respiratory specimens [45]. Serological tests can be divided into two categories: neutralization assays and binding antibody tests.

The former consists of plaque reduction neutralization tests, microneutralization, and surrogate virus neutralization tests [46]. The latter includes chemiluminescent immunoassays, enzyme-linked immunosorbent assays, electrochemiluminescence immunoassays, fluorescence immunoassays, protein microarrays, and immunofluorescence assays [46].

LFAs are a type of binding antibody tests which have been widely used for COVID-19 detection.

Jiao et al. have developed a gold nanoparticle-rapid diagnosis strip for detection of IgM response to the COVID-19 N protein. The bioreceptor was SARS-CoV-2 N protein immobilized on the test line and gold nanoparticle-labeled antihuman IgM was utilized as the detection reporter. Severe fever with thrombocytopenia syndrome virus, dengue virus, COVID-19-positive, and normal serum samples were employed to measure sensitivity (the likelihood of detecting the analyte when present) and specificity (the probability of the test to give a false positive) [18], which were reported as 100% and 93.3%, respectively [47].

An LFA approach for IgG detection was developed by Wan et al. through the use of immobilized N protein on the test line. They fabricated a conjugate pad with anti-human IgG-modified gold nanoparticles. The detection process was carried out by trapping the complex of IgG and anti-human IgG on the test line. Samples of patients with severe fever with thrombocytopenia syndrome and avian influenza A were employed to evaluate sensitivity and specificity, with 69.1% sensitivity and 100% specificity being reported for this assay [48].

Fang et al. have employed two colloidal gold chromatography paper assays for detecting anti-SARS-CoV-2 IgG and IgM directed towards S and N proteins. This assay involved anti-IgG and anti-IgM as the capturing reagents. Gold nanoparticles were modified with SARS-CoV-2 S and N proteins as indicators for the detection of the interaction between anti-IgG/IgM and the serological response. In the presence of anti-SARS-CoV-2 IgG and IgM, gold nanoparticle-labeled proteins are accumulated on the test line and create the optical signal. In this study, the created signal intensity is measured and converted to peak area by a portable reader. The sensitivity and specificity for anti-N IgG and IgM detection was obtained as 96%. The sensitivity and specificity for anti-S IgG were 95.9% and 96.1%, and for anti-S IgM were reported as 96.6% and 100% [49].

Another LFA approach was reported by Li et al. for detection of IgG and IgM against COVID-19. In this assay, if there is any IgM/IgG present in the sample, these antibodies can bind to a gold-labeled N protein antigen as the signaling agent and anti-IgM and anti-IgG as capturing agents, which were pre-coated on test lines. This assay was validated with a sensitivity of 95.85% and a specificity of 97.47% [50].

In another study, Anfossi et al. fabricated a multi-target lateral flow assay. This assay aimed at detecting of three classes of human anti-COVID-19 antibodies by using N protein and the staphylococcal protein A, which binds to Fab domains of IgA and IgM and also the Fc domain of IgG as bio-recognition elements and gold-labeled N protein as an optical signal reporter. They have claimed that the ability of recognizing total antibodies and using a double-antigen approach has enhanced the LFA’s sensitivity. This sensor was reported to display a fast result with high sensitivity and specificity of 94.6% and 100%, respectively [45].

This group also used a colorimetric LFA for detection of salivary anti-SARS-CoV-2 IgA. IgA has a time-dependent evolution and indicates the stage of infection. IgA antibodies in the sample bind to the nanogold-labeled anti-human IgA, then form a sandwich complex with coated N protein onto the test line. The test line will appear purplish red, indicating that the sample is positive for the COVID-19 IgA antibody. A noninvasive sample collection method and quantitative measurement are advantages of this assay [51].

Xu et al. applied gold nanoparticle-labeled S protein of SARS-CoV-2 for detection of IgG and IgM. If these antibodies are present in the sample, a formed antigen-antibody complex is captured by anti-human IgM or IgG on the test line and forms a visual band. This antibody analysis was tested by RT-PCR. They also compared the sensitivity of a single assay with combined IgG and IgM. The sensitivity for combined IgG and IgM (85.29%) detection is higher than single IgM (82.35%) and IgG (61.76%) detection [52].

A colorimetric-fluorescence-based LFA was developed by Wang et al. for diagnosis of anti-COVID-19 IgG and IgM. S protein-conjugated SiO_2_@Au@QD particles have been applied as the label. SiO_2_@Au@QD particles consist of SiO_2_ nanoparticle as a hydrophilic substrate carrying a layer of GNP and quantum dot (QD). The reported sensitivity of the fluorescence mode is 100 times higher than the colorimetric mode [53].

Another gold-labeled LFA was developed in two different designs for detection of COVID-19 antibodies. In these strategies, S protein was coated on the test line while gold-labeled goat anti-human IgG and gold-labeled S protein were used separately as signal reporters. Detection of total antibody content was performed, where gold-labeled S protein was used as a signal reporter. The results showed 100% specificity for both methods, and 30% and 90% sensitivity for the IgG test and total antibody detection, respectively [54].

In another study, Godfrey et al. have developed a GNP-based LFA to determine protected populations against COVID-19 by detection of RBD-ACE-2 neutralizing antibodies, consisting of more than 90% of COVID-19 neutralizing antibodies [55]. Neutralizing antibodies not only have a higher affinity than non-neutralizing ones [56], but they are also a reliable indicator of patient immunity. The designed LFA consists of two test lines and one control line. ACE-2 protein, recombinant RBD protein, and anti-chicken IgY antibody were coated on test line 1, test line 2, and the control line, respectively. GNP-conjugated RBD and GNP-conjugated chicken IgY were used as signal reporters. In the absence of neutralizing antibodies, GNP-conjugated RBD binds to ACE-2 and a red color appears on test line 1. In the presence of anti-RBD neutralizing antibodies, no red color can be seen on test line 1. In the presence or absence of neutralizing antibodies, GNP-conjugated chicken IgY binds to the anti-chicken IgY antibody, and the control line shows a red color. Total anti-RBD antibodies, neutralizing, and non-neutralizing antibodies form double-antigen sandwich test line 2 and create a red color. The reported specificity and sensitivity were 100% and 96%, respectively [55].

### 4.2. Detection of Antigen

An antigen-based lateral flow assay is an effective diagnostic tool that can meet the requirements of rapid detection in the shortage of laboratory instruments and experienced staff.

Such antigen rapid diagnostic tests (Ag-RDTs) with the potential for early diagnosis are less costly than RT-PCR methods and can be widely used for fast screening of an infected population. Until February 2022, 189 and 5 Ag-RDTs for SARS-CoV-2 detection have been approved by the EUA [57] and the WHO [58], respectively.

Vandenberg et al. reported an immunochromatographic assay for the detection of nucleocapsid (N) proteins. Firstly, monoclonal antibodies were constructed against full-length SARS N protein and evaluated with SARS-CoV-2 N protein. A conjugate pad consisting of anti-COVID-19 antibody-modified gold nanoparticles was used, and the test line contained anti-COVID-19 antibodies. In the presence of COVID-19 virus, the gold nanoparticle-conjugated antibody-SARS-CoV-2-capture antibody complex forms, and a red color appears on the test line. The diagnostic efficacy of the assay was assessed by RT-PCR. Nasopharyngeal samples containing respiratory virus and culture supernatant containing coronaviruses OC43, NL63, 229E, and HKU1, as well as SARS-CoV, were detected to validate specificity. No cross-reactivity was observed for viruses, except SARS-CoV. This sensor exhibited a sensitivity and specificity of 57.6% and 99.5%, respectively [59].

Gibson et al. have demonstrated a glycan-based LFA for detection of the S protein. It has been shown that sialic acid coordination with the S1 subunit of S protein is a necessity for SARS-CoV-2 entry into host cells. N-acetyl neuraminic acid, which is the predominant sialic acid found in human cells, was used as a capturing and detection reagent. For visual detection of SARS-CoV-2, sialic acid was conjugated to GNP. The binding specificity was tested by SARS S protein. The results revealed that this glycol-LFA has selectivity toward COVID-19 [60].

BIOCREDIT COVID-19 Ag (RapiGEN Inc., Gyeonggi-do, Korea) and Standard Q COVID-19 Ag (SD Biosensor, Gyeonggi-do, Korea) are also used to target SARS-CoV-2 antigen by gold-labeled monoclonal antibodies. The performance of these two assays was evaluated by RT-PCR. The results showed a sensitivity of 45% and 60% for BIOCREDIT COVID-19 Ag and SD Biosensor RAD kits, respectively [61].

In another investigation, monoclonal antibodies against the N protein and S protein of SARS-CoV-2 were developed. Evidence shows that there is a significant sequence conservation of S2 subunits among various coronavirus genera, while the sequence of N protein is conserved within the genus to a lesser extent. Hence, it was reasonable that the S antigen-monoclonal antibody showed cross-reaction with the S protein of HCoV-OC43, HCoV-NL63, and HCoV-HKU1. Therefore, a gold nanoparticle-based LFA based on N protein monoclonal antibodies was constructed. Although this assay can identify 0.1 ng/mL of N protein and does not show cross-reactivity with non-lethal coronaviruses, influenza A, influenza B, rubella virus, varicella zoster virus, and pyogenic bacteria, MERS-CoV N protein, and chicken IBV, it cannot differentiate between SARS-CoV and SARS-CoV-2 because both belong to the ß genus of coronaviridae [1].

Despite the reported cross-reactivity of S antigen-monoclonal antibodies with S proteins of other coronavirus heritage [1], Zhang et al. fabricated a specific gold-based immunochromatographic assay for detection of the SARS-CoV-2 spike protein. The detection limit of the assay is reported as 62.5 ng/mL and it can differentiate between SARS-CoV-2 and other members of the ß family, such as SARS-CoV, MERS-CoV, and influenza viruses [62]. The information of LFAs for detection of antigen and antibodies has been summarized in Table 2.

## 5. Limitations and Solutions

### 5.1. How Can the Specificity of LFAs Be Improved?

#### 5.1.1. Development of Specific Antibodies against SARS-CoV-2

Although LFAs for antibody detection mostly show high specificity, their performances were investigated mostly by SARS-CoV-2-positive and non-SARS-CoV-2 patients, while the cross-reactivity with SARS-CoV/MERS-CoV-positive human plasma was not evaluated [45,47,48,49,50,51,52,53,54].

In fact, the cross-reactivity from conserved antigens belonging to other viruses causes a high false-positive rate and a low specificity of immunological tests [63].

The previous observations indicate a strong cross-reactivity between samples of SARS-CoV-infected patients’ plasma and SARS-CoV-2 N protein [49]. In another study, Lv et al. have reported that the anti-SARS-CoV-2 and anti-SARS-CoV antibodies are both binding to the SARS-CoV-2 S protein [64].

As mentioned, this is because SARS-CoV-2, SARS-CoV, and MERS belong to the same β-CoV genera [65]. In one study, Schifferli et al. have compared six commercial antibodies for antigen interaction, and the results showed that antibodies raised against SARS-CoV-2 had cross-reactivity with SARS-CoV antigen [66].

Therefore, developing specific antibodies against SARS-CoV-2, without cross-reactivity with other coronaviruses, could be a solution for this problem. Park et al. used a phage display method to generate a specific single-chain variable fragment (scFv) against the COVID-19 N protein and applied it for developing an LFA. The developed LFA exhibited no cross-reaction with other coronaviruses [27].

#### 5.1.2. Deployment of Other Specific Bio-Recognition Elements

In addition to antibodies, other bioreceptors such as glycans have been used for specific detection of COVID-19. The previous study has revealed that the S1 subunit of coronaviruses binds to sialic acid on host cells. Thus, Gibson et al. have introduced sialic acid-based LFA for detection of SARS-CoV-2 S protein. The negligible homology of the sialic acid binding sequence of the S1 subunit between coronaviruses and the variety of glycan partners among different strains of host cells are two effective factors that allow the designed sensors to specifically detect the SARS-CoV-2 S protein and show selectivity over the SARS-CoV S protein [60].

### 5.2. How Can the Sensitivity of LFA Be Improved?

The analytical sensitivity of LFA should meet the clinical sensitivity [65]. Some commercialized gold-based rapid antigen/antibody detection tests for COVID-19 diagnosis are collected in Table 3 below.

There are studies that have evaluated the performance of commercial serological assays and observed a lack of sensitivity. For example, Ricco et al., in a systematic review and meta-analysis of ten studies, have reported that point-of-care assays for SARS-CoV-2 antibodies had a pooled sensitivity of 64.8% [67]. In another report, Zautner et al. have evaluated five commercial assays for detection of COVID-19-specific antibodies. The measured sensitivity and specificity ranged from 17% to 81.9% and 90.2% to 100%, respectively [68]. These studies reveal the low sensitivity of LFAs for detection of host antibodies.

According to previous studies, Ag-RDTs are nowhere near as sensitive as RT-PCR [61,69]. The reason is that Ag-RDTs lack an amplification step and can only be positive when a person has a high viral load, while RT-PCR tests amplify RNA viral load and thus can detect very limited presence of a virus [69]. For example, Denkinger et al. have evaluated the sensitivity and specificity of commercial Ag-RDTs. The systematic review and meta-analysis of 133 papers on Ag-RDTs published until 30 April 2021 showed that the pooled sensitivity and specificity compared to RT-PCR were 71.2% and 98.9%, respectively [70]. This result shows that due to the high false-negative rate, viral antigen tests have a low sensitivity [63]. So far, several strategies have been suggested to improve the sensitivity of LFAs, as summarized below.

#### 5.2.1. Platform of Assay

Lateral flow assays based on the double-antigen sandwich format are expected to increase the sensitivity by reducing non-specific antibody binding. In the traditional lateral assay format for antibody detection, the secondary antibody that is used as the signal reporter can bind to both specific and non-specific antibodies in the sample, which greatly decreases the sensitivity of the assay. In contrast, in the double-antigen sandwich format, the antigen works as a bioreceptor on the test line and as a signal reporter, and only specific antibodies are trapped on the test line. Therefore, the total and specific antibodies will be detected, which significantly increases the sensitivity [71]. Different studies have used the double-antigen sandwich strategy for detection of antibodies targeting COVID-19 [45,54,72].

In a further effort to increase sensitivity, the combination of two LFAs has been used for detection of anti-COVID-19 IgG and IgM in the same sample. Although this strategy did not have an effect on IgG detection, it could increase the sensitivity for IgM detection, i.e., 87.7% sensitivity was achieved for IgM detection by both LFAs, while 58.9% and 66.2% sensitivity were found for the kits alone [73].

#### 5.2.2. Infection Disease Stage

Another effective factor on sensitivity is the disease state. Studies have shown that IgG and IgM responses against SARS-CoV-2 are different from typical responses to antigen exposure [45]. For example, it has been observed that IgG production happens earlier than IgM for anti-N protein [48]. Another report showed that the sensitivity of rapid COVID-19 antibody (IgG or IgM) tests is lower than 80% at <20 days after symptom onset [56]. Weil et al. have also evaluated the sensitivity of six LFAs for COVID-19 antibody diagnostics. The result showed that sensitivity increased from 18.8% to 40.6% at 3 days after symptom emergence to 80.3–96.4% at >14 days after symptom onset [74]. In fact, the concentration of antibodies depends greatly on the time of the sampling. For example, studies showed that the amount of IgA antibody is higher than IgG in the early phase of disease [51].

Therefore, patients in the early or late phase of the infection and individuals with a weak immune response do not have a sufficient amount of antibodies (IgG or IgM) and are prone to false-negative results [67].

Ag-RDT sensitivity also depends on the disease onset time. The highest sensitivity is achieved when a person is most infectious and sensitivity significantly drops during incubation and the post-infectious period [69]. Hence, the viral load influences the sensitivity of the LFA.

For instance, Vandenberg et al. fabricated the COVID-19 Ag Respi-Strip and showed that sensitivity of the rapid assay is higher for health workers’ samples with a higher SARS-CoV-2 load [59]. In another study, Lindner et al. reported that the more viral load in a COVID-19 patient’s sample, the more sensitivity gains [75].

For further experiments, the performances of two commercialized rapid antigen tests were evaluated in samples with different viral loads. For the BIOCREDIT COVID-19 Ag and the SD Biosensor RAD kits, respectively 45% and 60% sensitivity were seen in normal viral load samples, while this was raised to 60% and 77% sensitivity in high viral load samples [61]. These findings are perhaps not so surprising, but they show that an appropriate timing of sampling is a contributing factor in assessing the sensitivity.

#### 5.2.3. Target Concentration

Signal intensity depends on the analyte concentration. Evidence shows that the signal generated by total antibodies is higher than the one produced by single antibodies, which in turn causes higher analytical sensitivity. Anfosi et al. achieved 94.6% sensitivity by developing an LFA for detection of three classes of antibodies (IgG, IgM, and IgA) instead of for individual antibodies [45]. The sensitivity of an LFA for detection of IgG and total antibodies specific to SARS-CoV-2 were compared by Cui et al. The results showed that the sensitivity for detection of single antibodies (30%) is lower than that for total antibodies (90%) [54]. Xu et al. also compared the sensitivity for detection of combined antibodies (IgG and IgM) with single antibodies (IgG or IgM) targeting COVID-19. The results again showed higher sensitivity for combined antibody detection [52].

#### 5.2.4. Sample Collection Method

Another effective factor on the sensitivity of assays is the sample collection procedure. WHO-listed SARS-CoV-2 Ag-RDTs use anterior nasal swab and nasopharyngeal/oropharyngeal swab samples. These sampling methods benefit from having a well-trained technician to correctly handle the sampling. It has been shown that self-administered Ag-RDTs have a lower sensitivity (58%) compared to that achieved by trained technicians (79%) [69]. Ideally, a user-friendly sampling technique which obviates the need for a professional staff and that achieves high sensitivity can be developed.

#### 5.2.5. Sample Preparation Method

The sample preparation procedure is a step with a large effect on the LFA performance. This step is needed to remove the matrix components and also to concentrate the analytes. The use of a carrier with immobilized receptor molecules on it can be an efficient method to capture the analyte from the sample. In this way, analytes are concentrated, and the sensitivity can increase. Wan et al. used streptavidin-coated magnetic beads to separate COVID-19 RNA from the sample. After RNA extraction, the cDNA was obtained and then amplified by a TAG-containing forward primer and a biotinylated reverse primer. The biotinylated PCR product was separated by magnetic beads and detection was performed by the colorimetric method [10].

Target separation was carried out for quantitative measurement of IgG and IgM by a DNA-assisted nanopore sensor. For this purpose, IgG and IgM antibodies against COVID-19 were captured with N protein-functionalized magnetic beads. Captured immunoglobulins were quantitatively detected by a nanopore biosensor [8].

#### 5.2.6. Proper Orientation of Bioreceptors

The proper orientation of bio-recognition elements with respect to the relevant surface of a membrane or reporter molecule can increase the accessibility of epitopes and improve the efficiency of immunosensors. Ravera et al. applied a computational model to predict the orientation of the receptor-binding domain (RBD) of the SARS-CoV-2 S protein on a surface [76]. Different strategies such as indirect binding can be used to gain oriented immobilization. For example, Nichols et al. coated the test line with polystreptavidin to properly immobilize biotinylated antibodies for capture and detection of SARS-CoV-2 virus in an assay [65]. Another method for indirect binding is the utilization of the Staphylococcal protein A with the capability of binding to the Fc domain of IgG and the Fab domain of IgM and IgA. For instance, Anfossi et al. have applied the Staphylococcal protein A as the capturing reagent on the test line for detection of total antibodies targeted against COVID-19. The trapped antibodies on the test line can then have the proper orientation to bind the reporter molecule [45].

#### 5.2.7. Proper Concentration of the Bioreceptor

Conditions of the reaction media can affect the performance of the LFA. Proper concentration of receptors on the test line is an effective factor on the cut-off level (threshold of disparity between negative and positive samples). For example, Jiao et al. have evaluated different concentrations of nucleoprotein on the test line to gain the best signal-to-noise ratio for detection of IgG and IgM against SARS-CoV-2. The result showed that at concentrations lower than 1 mg/mL of N protein, the red color on the test line is not easily detectable, and at higher concentrations, the correlation of response is not as good as at 1 mg/mL [47,48]. The concentration of printed antibodies on the test line was also evaluated by Wang et al. The results showed that the highest signal-to-noise of fluorescence intensity for COVID-19 antibody detection was generated by 0.9 and 1.2 mg/mL of coated anti-human IgM and IgG on the test line [53]. The concentration of covered antibodies on GNP is also of great importance [47,48]. Thus, different concentrations of antibodies were added to determine the optimal amounts of antibodies on the gold nanoparticle.

#### 5.2.8. Membrane Properties

Biomolecule trapping in the membrane pores can decrease binding efficiency. A procedure for LFA membrane blocking is necessary to address this issue. Wang et al. evaluated the use of different concentrations of BSA and showed that the absence of BSA blocking and excessive blocking resulted in false-positive results [47,48].

The movement of the reagents through the test strip is affected by the pore size of the membrane. Different types of nitrocellulose membranes (NC140 and NC95) were compared for developing dual-mode LFA for detection of SARS-CoV-2-specific IgG and IgM. The result showed that NC140 with a smaller pore size generated a higher signal [53].

#### 5.2.9. Signal Reporter Properties

The recorded signal intensity of the gold nanoparticle (GNP) as a signal reporter is an important determinant for the sensitivity of the assay. The limited sensitivity of gold nanoparticle-based LFAs is due to the low GNP capture rate [77]. Additionally, GNP parameters such as the shape and size can have an effect on the detection limit. Gibson et al. have used N-acetyl neuraminic acid-conjugated gold nanoparticles to capture the SARS-CoV-2 S1 protein. The impact of different sizes of gold nanoparticles (16, 35, 55, 70 nm) on the binding was evaluated. The results showed that 35 nm-diameter GNPs afforded a superior signal-to-noise ratio compared to 16 nm particles, and also that larger particles did not further improve the signal intensity [60].

However, the increase in the sensitivity can normally be achieved through enlargement of gold nanoparticles. The larger the GNPs are, the higher scattering component of extinction coefficient the GNPs have. Dzantiev et al. have applied a GNP-based LFA for detection of the receptor-binding domain (RBD). Three enhancement approaches were utilized for raising the sensitivity, namely silver enhancement, galvanic replacement, and gold enhancement, which yielded, respectively, 8, 61, and 488 times higher sensitivity than conventional LFAs with GNPs [78].

#### 5.2.10. Read-Out Strategy

Generally, LFA is a transducer-free method, and results are assessed by the naked eye. However, conversion of qualitative LFA results to quantitative data provides the possibility of collecting information and can increase the sensitivity. For example, Scully et al. have applied laser optical analysis to enhance sensitivity 100 times and quantify the amount of SARS-CoV-2-specific antibodies [79]. In another study, Anfossi et al. have used a portable imaging device to measure the anti-COVID-19 IgA concentration in saliva [51]. Fang et al. have developed a homemade portable reader for objective detection of IgG- and IgM-targeted COVID-19 [49]. Quantitative measurement of anti-COVID-19 antibodies has been performed by integrating LFA and the portable optical spectrometer [80].

**Figure 2:**
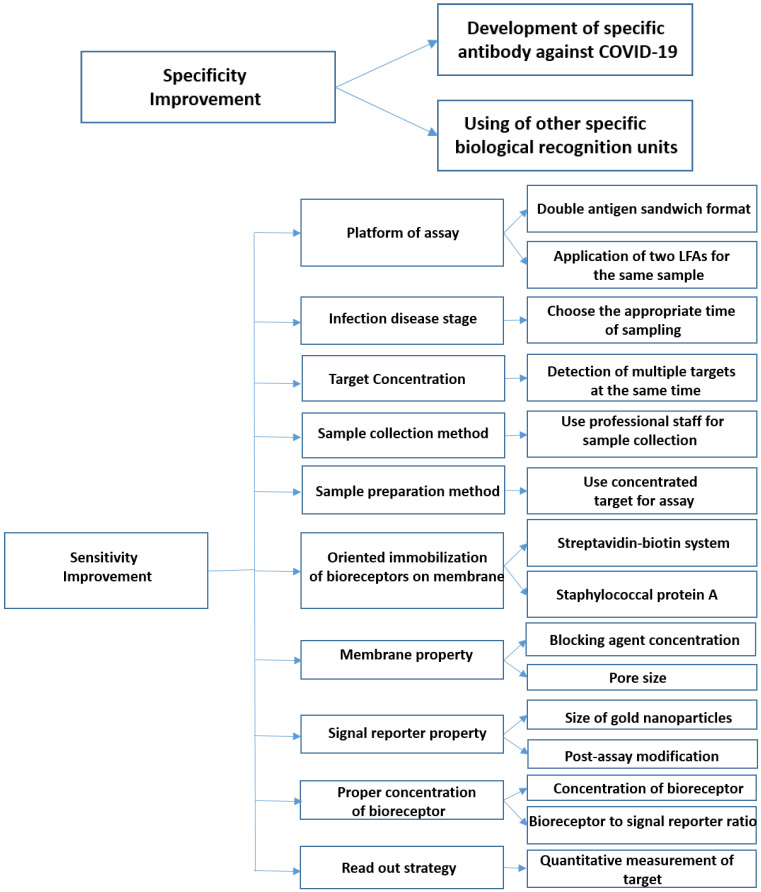
Approaches for improving the performance of LFAs. These methods can be divided into two groups: improvement of sensitivity and improvement of specificity.

These above-mentioned methods for improving the specificity and sensitivity of lateral flow assays have been summarized in Figure 2.

## 6. Conclusions

Lateral flow assays are inexpensive, easy-to-operate, portable, and instrument-free point-of-care options that have been widely used in multiple phases of the COVID-19 pandemic. Their ease of use makes them a suitable choice for screening at public venues such as airports, shopping centers, and sport stadiums. Rapid diagnosis of virus-specific proteins and host antibodies has been utilized to detect active and previous infections in turn. Although regulatory bodies have approved many of these assays, cross-reactivity and low sensitivity remain the two main weaknesses associated with them. Some methods such as CRISPR-based tests for RNA detection have been able to achieve higher sensitivity and specificity, which comes at the expense of more complexity and the need for additional instrumentation. Additionally, they have a higher price point per analysis compared with the majority of rapid-access tests.

Utilization of gold nanoparticle-based LFAs for COVID-19 detection has shown a fine balance of complexity and high sensitivity. These tests are not as complex as an RNA-based assay and several approaches have been introduced to improve their sensitivity and specificity. On several fronts, significant progress has been made in the performance of GNP-based LFAs for COVID-19 diagnosis. However, researchers continue to push these boundaries for an improved performance. For example, analyte pre-concentration methods such as aqueous two-phase systems (ATPSs) [81], dialysis-based concentration [82], and paper-based iso-tachophoresis (ITP) [83] have been introduced to enhance the sensitivity of the LFAs. Additionally, pre-concentration of transferrin by ATPSs has been reported to improve the detection limit of LFAs 100-fold [81].

## 7. Future Perspectives

Improving antibody-antigen affinity is a vital contributing factor to enhance the sensitivity of LFAs. A way to proceed may be use of the variable region of camelid heavy-chain antibodies (VHHs) as single-domain antibodies which can be purified against specific epitopes through screening processes.

Several studies have worked on the production of VHH against SARS-CoV-2 [84,85,86]. For example, Saelens et al. have produced a VHH-human immunoglobulin Fc fusion protein against the RBD of spike proteins. The produced nanobody not only shows sub-nanomolar affinity toward SARS-CoV-2 but also binds to other circulating variants such as B.1.1.7. and B.1.351 [84].

Therefore, such nanobodies with high affinity can be exploited to obtain sensitive detection of SARS-CoV-2 and other variants of concern in the LFA format.

Optimizing the target concentration by exploiting multiple targets is another aspect that is important for the improvement of LFA performance. For example, LFAs that measure total antibodies show higher sensitivity than those that measure single ones. Along these lines, Ventura et al. have enhanced the sensitivity of GNP-based biosensors by detecting three surface proteins (S, E, and M). The measured detection limit is close to RT-PCR, so a similar approach can be used in an LFA platform in order to enhance sensitivity for detection of SARS-CoV-2 [3].

While such avenues for improving sensitivity and specificity have been introduced, some further challenges still remain for GNP-based LFAs, such as improving the reproducibility of the gold nanoparticle synthesis and integration of assays with, e.g., a smart phone read-out, which are yet to be overcome. The combination of these initiatives may pave the way to a universal rapid testing solution that is readily available and accepted worldwide.

The many leaps forward already taken in the development of GNP-based assays shows the great potential of the methodology, and we foresee that the area of application can extend far beyond the current need for SARS-CoV-2 diagnosis to a range of other infections of concern in the future.

## Figures and Tables

**Table 1:** Different targets that have been applied for COVID-19 diagnosis include detection of viral RNA, products of immunological reactions, i.e., IgA, IgG, and IgM antibodies and interleukin 6 (IL-6), as well as viral proteins E, M, S, and N.

Biosensors for COVID-19 Diagnosis			
Targets		Methods	Ref.
RNA		Colorimetric-based assay	[4]
		Fluorescence-based assay	[10]
Immunological reaction	IgG, IgM, IgA	Colorimetric-based assay	[6]
		Electrical-based sensor	[8]
		Fluorescence-based assay	[11]
		Optical fiber sensor	[12]
	IL-6	Colorimetric-based assay	[5]
Viral proteins		Colorimetric-based assay	[3]
		Electrical-based sensor	[7,9]
		Optical fiber sensor	[12]
		Magnetic nanoparticle-based biosensor	[13]
		Surface Plasmon Resonance-based sensor	[14]
		Electrochemical immunosensor	[15]
		Colorimetric-based assay	[16]

**Table 2:** Overview of gold-based lateral flow assays for detection of antigen and antibodies of SARS-CoV-2.

Target	Bioreceptor on Test Line	Gold-Labeled Reporter	Specificity	Sensitivity	Sample	Ref.
Anti-N IgM	N Protein	Anti-human IgM	93.3%	100%	Serum	[47]
Anti-N IgG	N Protein	Anti-human IgG	100%	69.1%	Serum	[48]
Anti-N IgG	Anti-human IgG	N Protein	96%	96%	Serum	[49]
Anti-S IgG	Anti-human IgG	S-RBD protein	96.1%	95.9%	Serum	[49]
Anti-S IgM	Anti-human IgM	S-RBD protein	100%	96.6%	Serum	[49]
Anti-N IgG and IgM	Anti-human IgG and IgM	N protein	97.47%	95.85%	Serum	[50]
Anti-N IgG/IgM/IgA	N protein	N protein	94.6%	100%	Serum	[45]
Anti-N IgA	N protein	Anti-human IgA	-	-	Saliva	[51]
Anti-S IgG	Anti-human IgG	S Protein	-	61.76%	Serum	[52]
Anti-S IgM	Anti-human IgM	S Protein	-	82.35%	Serum	[52]
Anti-S IgG/IgM/IgA	S Protein	S Protein	100%	90%	Serum	[54]
Neutralizing Antibodies	ACE-2	RBD	100%	96%	Whole blood	[55]
N protein	Anti-COVID-19 antibody	Anti-COVID-19 antibody	99.5%	57.6%	Nasopharyngeal sample	[59]
S protein	N-acetyl neuraminic acid	N-acetyl neuraminic acid	-	-	-	[60]
N protein	Anti-COVID-19 antibody	Anti-COVID-19 antibody	-	-	Nasopharyngeal and oropharyngeal samples	[1]
S protein	Anti-COVID-19 antibody	Anti-COVID-19 antibody	-	-	Recombinant S protein	[62]

**Table 3:** Commercialized gold-based lateral flow assay for detection of antigen and antibodies of SARS-CoV-2.

Company	Product	Sample Source	Target	Storage Condition
Jiangsu Well Biotech Co., Ltd. (Changzhou, China)	Orawell IgM/IgG Rapid Test	Serum	IgM and IgG	2–8 ℃
Xiamen Biotime Biotech-nology Co., Ltd. (Xiamen, China)	BIOTIME SARS-CoV-2 IgG/IgM Rapid Qualitative Test	Serum	IgM and IgG	-
Megna Health, Inc. (Exton, PA, USA)	Rapid COVID-19 IgM/IgG Combo Test Kit	Serum	IgM and IgG	4 to 30 °C
Access Bio, Inc. (Somerset, NJ, USA)	CareStart COVID-19 Antigen Test	Nasopharyngeal swab (NPS)	Nucleocapsid Antigen	1 to 30 °C
Beijing Wantai Biological Pharmacy Enterprise Co., Ltd. (Beijing, China)	Rapid test for SARS-CoV-2 Antigen	Nasopharyngeal swab (NPS)	Nucleocapsid Antigen	-
Zhuhai Livzon Diagnostics (Guangdong, China)	The Diagnostic Kit for IgM/IgG Antibody to Coronavirus (SARS-CoV-2)	serum, plasma, venous whole blood	IgM and IgG	2 to 30 °C
Zhuhai Livzon Diagnostics (Guangdong, China)	Livzon Rapid Test for SARS-CoV-2 Antigen	Nasopharyngeal swabs, oropharyngeal swabs	-	2 to 30 °C
Assure Tech Co. (Hangzhou, China)	Assure COVID-19 IgG/IgM Rapid Test Device	serum, plasma, venous whole blood	IgG and IgM	-
Beijing Diagreat Biotechnologies (Beijing, China)	2019-nCoV IgG/IgM Antibody Rapid Test Kit	Blood	IgG and IgM	-
Biolidics (Singapore)	Rapid test kit for COVID-19-IgG/IgM Antibody Detection Kit	venous whole blood/serum/plasma	IgG and IgM	2–8 °C
Sugentech (Daejeon, Korea)	SGTi-flex COVID-19 IgM/IgG	venous whole blood/serum/plasma	IgG and IgM	-
SD Biosensor (Gyeonggi-do, Korea)	STANDARD Q COVID-19 IgM/IgG Duo	venous whole blood/serum/plasma	IgG and IgM	2 to 30 °C
SD Biosensor (Gyeonggi-do, Korea)	STANDARD Q COVID-19 Ag Test	Nasopharyngeal swab	-	2 to 30 °C
BioVendor R&D (Brno, Czech Republic)	BIOCREDIT COVID-19 Ag Detection Kit	Nasopharyngeal swabs	-	-
